# Stillbirth after COVID-19 in Unvaccinated Mothers Can Result from SARS-CoV-2 Placentitis, Placental Insufficiency, and Hypoxic Ischemic Fetal Demise, Not Direct Fetal Infection: Potential Role of Maternal Vaccination in Pregnancy

**DOI:** 10.3390/v14030458

**Published:** 2022-02-23

**Authors:** David A. Schwartz

**Affiliations:** 1950 Grace Arbor Court, Atlanta, GA 30329, USA; davidalanschwartz@gmail.com

**Keywords:** COVID-19, SARS-CoV-2, pregnancy, placenta, placental insufficiency, stillbirth, intrauterine fetal demise, SARS-CoV-2 placentitis, vaccination, massive perivillous fibrin deposition, trophoblast necrosis, chronic histiocytic intervillositis

## Abstract

Stillbirth is a recently recognized complication of COVID-19 in pregnant women. Other congenitally transmitted infections from viruses, bacteria and parasites can cause stillbirth by infecting fetal organs following transplacental transmission of the agent from the maternal bloodstream. However, recent research on pregnant women with COVID-19 having stillbirths indicates that there is another mechanism of stillbirth that can occur in placentas infected with SARS-CoV-2. In these cases, viral infection of the placenta results in SARS-CoV-2 placentitis, a combination of concurrent destructive findings that include increased fibrin deposition which typically reaches the level of massive perivillous fibrin deposition, chronic histiocytic intervillositis and trophoblast necrosis. These three pathological lesions, in some cases together with placental hemorrhage, thrombohematomas and villitis, result in severe and diffuse placental parenchymal destruction. This pathology can involve greater than one-half of the placental volume, averaging 77% in the largest study of 68 cases, effectively rendering the placenta incapable of performing its function of oxygenating the fetus. This destructive placental process can lead to stillbirth and neonatal death via malperfusion and placental insufficiency which is independent of fetal infection. Fetal autopsies show no evidence that direct infection of fetal organs is contributory. Because all mothers examined have been unvaccinated, maternal vaccination may prevent viremia and consequent placental infection.

## 1. Introduction

Human outbreaks from two coronavirus species prior to the coronavirus disease 2019 (COVID-19) pandemic were the cause of adverse obstetrical outcomes [1,2,3]. Both severe acute respiratory syndrome coronavirus (SARS-CoV) and Middle East respiratory syndrome coronavirus (MERS-CoV) have occurred in pregnant women, causing maternal and perinatal morbidity and mortality [1,2,3,4,5,6,7]. In December 2019 a newly emergent coronavirus, termed severe acute respiratory syndrome coronavirus-2 (SARS-CoV-2), resulted in a large outbreak of respiratory tract disease in Wuhan, China [8,9]. As SARS-CoV-2 spread globally, during the early stages of the COVID-19 pandemic there was great concern that the virus would be transmissible from infected mothers to their fetuses [1,2,3,10,11]. However, previous experience with SARS-CoV and MERS-CoV during pregnancy had not identified intrauterine transmission [1], and other respiratory RNA viruses demonstrated either non-existent or rare instances of maternal-fetal transmission [2]. Although initial reports from China indicated that pregnant women having COVID-19 were not transmitting the virus to their neonates, apprehension concerning the possibility of fetal infection and other adverse outcomes remained [11,12,13]. As large numbers of pregnant women became infected, increasing numbers of neonates testing positive for SARS-CoV-2 were reported from China and elsewhere [14,15,16,17,18]. Eventually, placental pathology analysis confirmed that maternal-fetal transmission from infected mothers was occurring in a small percentage of infants [19,20,21,22,23,24,25], making SARS-CoV-2 the newest congenitally transmissible agent to be described [26]. Raschetti and colleagues [19] estimated that 5.7% of cases of neonatal SARS-CoV-2 infection was transmitted via this mechanism.

Among the adverse obstetrical and perinatal outcomes observed associated with COVID-19 in pregnancy, stillbirth incidence was not initially believed to be increasing [27,28,29]. However, as new variants of the virus developed and spread, practitioners and hospitals experienced increasing numbers of stillborn fetuses, including a cluster of stillborn fetuses occurring in mothers with COVID-19 in Ireland [30] and a report from England showing increased risk for fetal death among a cohort of SARS-CoV-2-infected pregnant women [31]. In November 2021 the U.S. Centers for Disease Control and Prevention confirmed that the pregnant women having COVID-19 were at an increased risk for stillbirth, especially during the time of the SARS-CoV-2 B.1.617.2 (Delta) variant predominance [32].

Microbial agents that are contracted before or during pregnancy which can be transmitted to the fetus are termed TORCH agents (an acronym for Toxoplasma, Other, Rubella, Cytomegalovirus, Herpes). These infectious agents can cause stillbirth by passing from the maternal circulation into the placenta, where they cross the maternal-fetal interface to infect the fetus, typically resulting in organ damage and, in some cases, death. However, until recently it had not been determined how SARS-CoV-2 was causing stillbirth and early neonatal death in pregnant women with COVID-19. This communication describes the current evidence for the mechanism(s) of fetal demise from SARS-CoV-2, the newest TORCH virus.

## 2. SARS-CoV-2 Placentitis

In the beginning stages of the COVID-19 pandemic placentas were collected and analyzed to determine the potential for SARS-CoV-2 to cause placental damage and infect the fetus. Placental pathology had been a highly useful technique in understanding maternal-fetal transmission with other TORCH viruses, including Zika virus [33], Ebola virus [34], and others. However, the initial results from examining placentas from mothers with COVID-19 were variable and differed significantly between reports. Some studies revealed fetal or maternal vascular malperfusion [35,36], others showed both processes [37,38,39], and some described hemorrhage, clots and a spectrum of different inflammatory lesions including chronic histiocytic intervillositis, villitis, funisitis, and chorioamnionitis [37,38,39]. There were also reports of a lack of specific findings of COVID-19 in placentas from infected women [40,41], adding to the inconsistency of findings and conclusions of the pathology associated with SARS-CoV-2. A caveat of these disparate results was that the large majority of placentas evaluated had not been infected with SARS-CoV-2 and were from infants testing negative for the virus.

**Figure 1:**
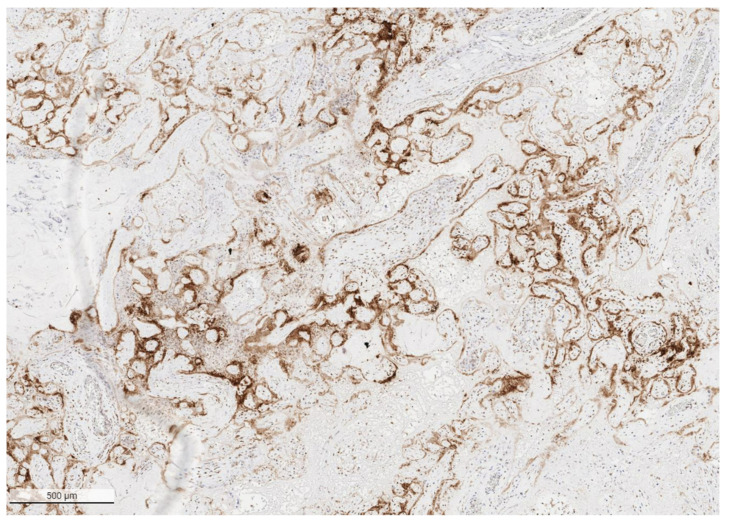
Low power image of placenta with SARS-CoV-2 placentitis from a stillborn fetus showing the pattern of positive staining of the villous syncytiotrophoblast for SARS-CoV-2 antigen. Antibody to SARS-CoV-2 spike protein, ×4.

**Figure 2:**
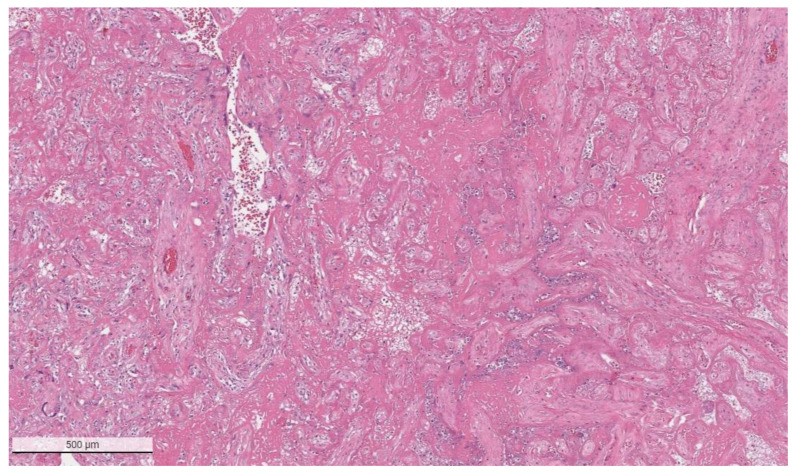
Massive perivillous fibrin deposition in a placenta from a stillborn fetus delivered to a mother with COVID-19. This placenta was positive for SARS-CoV-2 and had greater than 90% tissue destruction. Hematoxylin & eosin staining, ×4.

**Figure 3:**
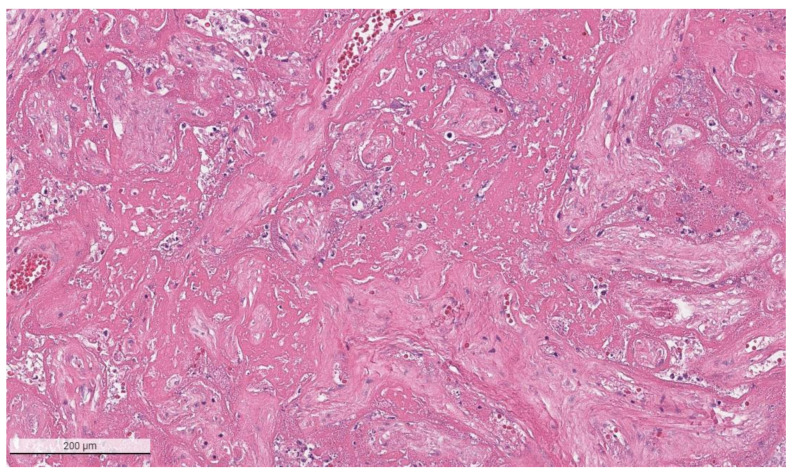
Higher magnification of a placenta with SARS-CoV-2 infection and SARS-CoV-2 placentitis. There is massive perivillous fibrin deposition with ischemic necrosis of the chorionic villi and villous trophoblast. There is complete obstruction of the intervillous space with fibrin which prevents maternal blood flow though this region of the placenta. Hematoxylin & eosin staining, ×10.

**Figure 4:**
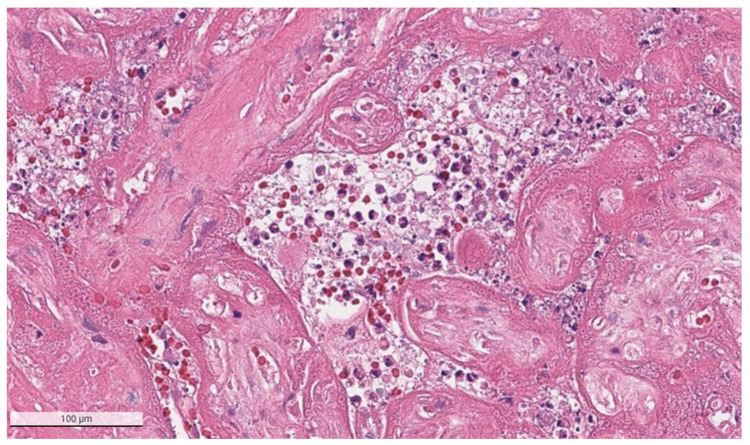
Chronic histiocytic intervillositis is present demonstrating histiocytes within the remnants of the intervillous spaces in a placenta with massive perivillous fibrin deposition and trophoblast necrosis. This stillborn fetus was delivered to a mother with COVID-19. The syncytiotrophoblast of this placenta was strongly positive for SARS-CoV-2 antigens using immunohistochemistry. Hematoxylin & eosin staining, ×20.

**Figure 5:**
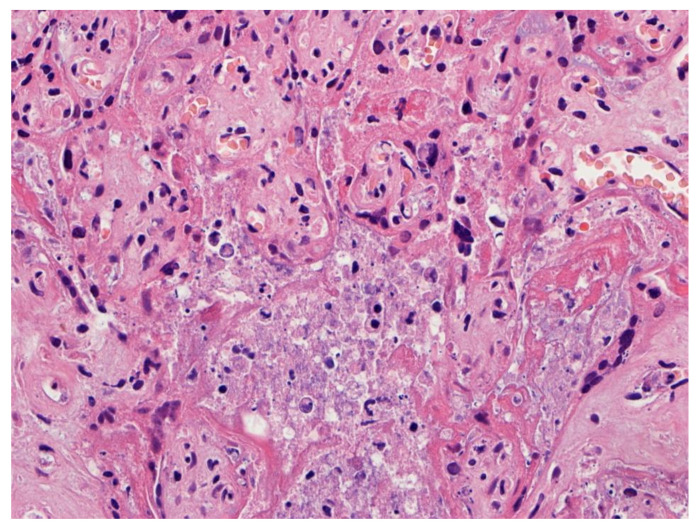
This placenta was infected with SARS-CoV-2 and demonstrates necrosis of the villous trophoblast. The intervillous space is complete obstructed with fibrin, remnants of histiocytes, and cellular and karyorrhectic debris, preventing maternal blood flow and oxygen delivery to the villi. Hematoxylin & eosin staining, ×20. Photograph courtesy of Fabio Facchetti, MD, PhD, Pathology Unit, Department of Molecular and Translational Medicine, Università degli Studi di Brescia (Brescia, Italy).

The placental pathology effects of SARS-CoV-2 became clearer after Schwartz and Morotti reported a study of infected maternal-fetal dyads having placentas that were positive for SARS-CoV-2 using immunohistochemistry with antibodies to viral antigens or detection of coronavirus nucleic acid by RNA in situ hybridization [23,42] (Figure 1). The infected placentas had a unique and highly repetitive pattern of pathological findings compared with the variable pathology findings in uninfected placentas [23]. Additional reports found that placentas testing positively for SARS-CoV-2 were typically characterized by a spectrum of destructive findings that included villous trophoblast necrosis, chronic histiocytic intervillositis and increased fibrin up to the level of massive perivillous fibrin deposition [20,21,24,43,44,45,46,47] (Figure 2, Figure 3, Figure 4 and Figure 5). A cohort study of 11 still- and liveborn babies having placentas infected with SARS-CoV-2 confirmed these findings to be risk factors for intrauterine viral transmission, perinatal morbidity and mortality [48]. Following examination of additional placentas, the term SARS-CoV-2 placentitis was introduced to refer to the triad of findings of chronic histiocytic intervillositis, perivillous fibrin deposition which typically reached the level of massive perivillous fibrin deposition, and villous trophoblast necrosis occurring in a placenta delivered from a mother with COVID-19 [49]. It was also found that among placentas infected with SARS-CoV-2 the syncytiotrophoblast is the most common cell type to be involved, although other cells including cytotrophoblast, Hofbauer cells, and villous stromal and endothelial cells could also be positive [26,50,51].

## 3. Placental Insufficiency and Stillbirth

The placenta is a dynamic fetal organ that is essential for fetal growth and development. In order to meet the changing demands and stresses of the growing fetus and intrauterine environment, it has the capability to alter its architecture, vascularity, physiology and signaling throughout the gestational period. The placenta serves many functions, but foremost among them is its ability to mediate oxygen transfer to the developing fetus from the maternal bloodstream. This process is only partially understood, and there remain many unanswered questions regarding the mechanisms and efficacy of transplacental oxygen transfer in placentas under normal conditions as well as with disease [52]. The human placenta has a hemochorial circulation in which oxygenated maternal blood coming from the spiral (decidual) arteries of the uterus enters the placental intervillous space and bathes the chorionic villous trees containing fetal vessels [53]. A requirement for this mechanism of efficient oxygen extraction from maternal blood is that the velocity and pattern of blood flow within the intervillous space always preserves a concentration gradient between the maternal and fetal circulations throughout the entire placenta, and that the intervillous space remain unobstructed to permit the free flow of maternal blood. In addition, the approximately 50 chorionic villous trees and their branches, including the tertiary villous capillary bed, must be intact and functional. Oxygen diffusion from maternal blood to fetal blood occurs at specialized adaptive capillary structures known as vasculo-syncytial membranes, consisting of fetoplacental endothelium and syncytiotrophoblast. This complex anatomic and physiological process of oxygen diffusion is subject to disruption at many points should a disease process interfere with perfusion or tissue integrity.

Because the placenta is designed to maximize the transfer of gases and nutrients between the mother and fetus, when the system fails a wide range of maternal and fetal complications can develop. In particular, there are specific placental pathology abnormalities that can result in reduction or obstruction of maternal and/or fetal blood flow into and through the placenta or result in irreversible injury to the chorionic villous tree. Depending on the etiology, duration, and intensity of these pathologic process(es), significant maternal or fetal vascular malperfusion can ensue, which in severe cases can result in placental insufficiency, fetal hypoxia and stillbirth.

Examination of the placenta has been found to be a highly useful method for identifying the cause(s) of intrauterine fetal demise because placental abnormalities are the most commonly identified etiology [54,55,56,57]. These abnormalities include pathological conditions that produce malperfusion, as well as specific histologic abnormalities including chronic histiocytic intervillositis and massive perivillous fibrin deposition [54], that can produce placental insufficiency. Unified criteria for the diagnosis of placental insufficiency do not exist; however, there is general agreement that it is a pathological process characterized by ongoing and continual deterioration in placental functioning. This causes decreasing placental transfer of maternal-derived oxygen and nutrients to the fetus, resulting in intrauterine fetal hypoxia, hypoxemia, and acidosis [58,59,60,61].

Prior to the COVID-19 pandemic, placental insufficiency was typically associated with abnormalities of perfusion which were either fetal or maternal in origin. These malperfusive conditions result from a variety of processes including underlying maternal diseases (hypertensive diseases of pregnancy, diabetes, maternal coagulopathies), fetal diseases (fetal coagulopathies, umbilical cord accidents, abnormal placental development and implantation), environmental exposures (cigarette smoking), abruptions, and placental conditions such as maternal floor infarcts, massive perivillous fibrin deposition, villitis of unknown etiology, failure of physiological conversion of decidual arteries, and others. Viral infections caused by TORCH agents typically do not produce placental insufficiency but instead exert their effects on the fetus after passing through the maternal-fetal interface at the placenta, entering the fetal bloodstream, reaching and directly infecting fetal organs [62,63,64]. There has not been a viral infection of humans ever reported that consistently and characteristically produces placental insufficiency to the extent of causing intrauterine fetal demise or perinatal death.

## 4. COVID-19 Infection and Stillbirth

During the early stages of the global pandemic in 2020, the effects of SARS-CoV-2 infection on stillbirths showed mixed results. A study conducted at two major Philadelphia hospitals showed no increase of stillbirths associated with COVID-19 [65]. In the United Kingdom, an analysis of stillbirth data derived from the National Health Service hospital admissions did not show any rise in incidence above the pre-pandemic baseline figures [66]. In nationwide study in Sweden showed no increase in the risk of stillbirth in the initial months of the pandemic up to May 2020 [67]. A study performed in the Castilla y León region of Spain also failed to demonstrate any increase of fetal demise up to 21 June 2020 [27].

In contrast, other reports contradicted these findings and identified elevated rates of stillbirth when compared with pre-pandemic levels [68]. These early increases, however, appeared to be an indirect effect of the pandemic associated with lockdowns and restricted access to antenatal care. In another report from England, Khalil et al. found that the stillbirth rate rose from 1.7% in the pre-pandemic period to 7% from 1 February to 14 June 2020. However, the mothers were asymptomatic, and results of autopsy and placental pathology examination were not consistent with SARS-CoV-2 infection, indicating that none of the stillbirths had occurred in women with COVID-19. The reported increase was attributed to fewer prenatal visits because of hesitancy to visit health care facilities and changes in referral patterns [69]. In Italy, the first country in Europe affected by COVID-19, there was a 3-fold increase in stillbirths in March to May 2020 which was believed to result from the lockdown and reduced visits to healthcare providers and hospitals [70]. Similar trends of increased rates of stillbirth associated with regional lockdowns during the early pandemic were seen in Nepal and other countries [71].

As the new variant strains of SARS-CoV-2 spread throughout the world in 2020 and 2021 there were anecdotal observations by clinicians and pathologists of increasing numbers of stillbirths that were associated with maternal COVID-19, but their causal relationship with coronavirus infection remained uncertain. Then, in the early months of 2021, physicians in Scotland noted a cluster of six stillbirths and a miscarriage that occurred within a few weeks after pregnant women developed COVID-19 from the B.1.1.7 variant [72,73,74]. This observation was followed in May 2021 with a report of 3527 pregnant women having confirmed COVID-19 in England in which mothers having SARS-CoV-2 infection had higher rates of fetal death than did uninfected mothers [31].

On 26 November 2021, the etiological association of SARS-CoV-2 infection with stillbirth was confirmed in a report from the U.S. Centers for Disease Control and Prevention [32]. A population-based study 1,249,634 delivery hospitalizations occurring from March 2020 to September 2021 demonstrated that pregnant women with COVID-19 had an increased risk for stillbirth compared with uninfected women, and that the strength of this of association was greatest during the period of the SARS-CoV-2 B.1.617.2 (Delta) variant predominance [71]. Recently, it has been found that coronavirus-infected women who were unvaccinated were far more likely than the general pregnant population to have a stillborn infant or one that dies in the first month of life [75,76].

**Figure 6:**
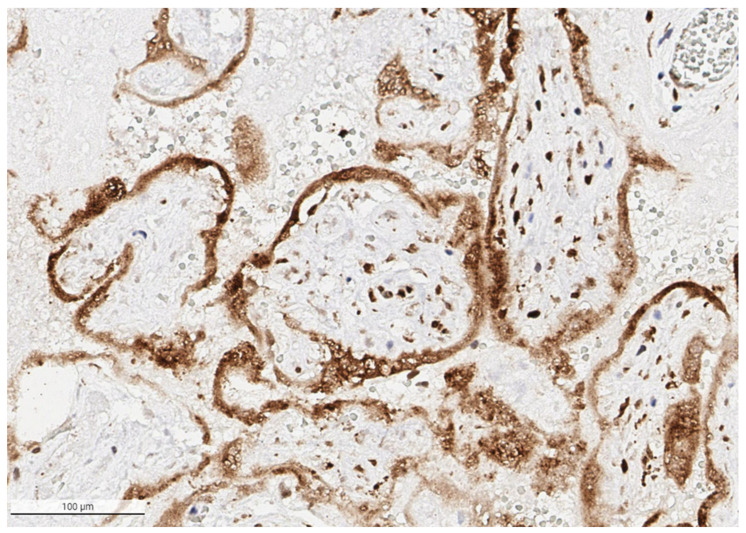
The syncytiotrophoblast is strongly positive for SARS-CoV-2 antigen in the placenta from a stillborn fetus delivered at 35 weeks 4 days to a mother with COVID-19 infection. Cells in the villous stroma can also be seen as staining positive for the virus. Antibody to SARS-CoV-2 spike protein, ×20.

Examination of placentas infected with SARS-CoV-2 from stillborn fetuses has been enlightening, significantly adding to the understanding of the mechanism(s) for intrauterine fetal demise. These studies have largely been made possible by the development of techniques for the tissue localization of SARS-CoV-2 in the placenta including immunohistochemistry to detect viral antigens and RNA in situ hybridization to identify viral nucleic acid [25,42,77] (Figure 6). Unfortunately, in the large majority of these cases the specific strain of SARS-CoV-2 was not identified. Following initial case reports of the pathology features of placentas infected with SARS-CoV-2 [19,20,21,22,24], Schwartz et al. in 2020 reported that the placental pathology findings from five stillborns having SARS-CoV-2 infections of the placenta were similar and included chronic histiocytic intervillositis, necrosis of villous trophoblast and positivity of syncytiotrophoblast for SARS-CoV-2 using immunohistochemistry or RNA in situ hybridization [48]. Extensive intervillous fibrin deposition, involving up to 70% or more of the placental tissues in one case, was present in the placentas from all 5 fetuses. Following this report, additional case reports of the placental pathology from stillborn fetuses delivered to mothers with COVID-19 were reported that showed all the placentas to be markedly abnormal and extensively involved with similar destructive features–increased or massive intervillous fibrin deposition, villous trophoblast necrosis, and chronic histiocytic intervillositis [78,79,80,81,82,83,84,85,86,87,88,89,90]. In their report of a stillbirth from a mother having COVID-19 at 30 weeks 4 days gestation in Bratislava, Biringer et al. [80] believed the cause of death to be acute placental insufficiency based upon their findings of placental destruction due to SARS-CoV-2 placentitis and an autopsy revealing fetal organs to have no morphological abnormalities. In another case of probable placental insufficiency causing stillbirth, di Gioia et al. [79] described a mother with COVID-19 from Rome who had a stillborn fetus at 36 weeks and 1 day of gestation. The placenta demonstrated extensive involvement by regions of hemorrhagic or ischemic necrosis with central and peripheral villous infarctions, and thrombosis of several fetal and maternal vessels with luminal fibrin and platelet depositions. There was immunohistochemical positivity of the syncytiotrophoblast for SARS-CoV-2 spike protein, and the autopsy showed no evidence of viral infection. Another mother with COVID-19 and stillbirth from Bratislava was described by Babal and colleagues [83]. In this 38-week gestation stillbirth with extensive SARS-CoV-2 placentitis and placental tissue destruction, they ascribed the cause of fetal death to fet ischemia. An autopsy revealed PCR positivity in lung, nasopharynx, and umbilical cord for SARS-CoV-2 RNA, and rare cells in the lung positive for the virus using immunohistochemistry and RNA in situ hybridization, but as there were no significant gross or microscopic changes in the fetal organs the authors believed that the viral positivity in the fetus was without significant impact in causing death. A stillbirth occurring from a mother with COVID-19 at 34 weeks 5 days from Rio de Janeiro was described by Marino et al. [86]. This placenta was severely affected by lesions of SARS-CoV-2 placentitis as well as from intervillous and subchorionic thrombi; SARS-CoV-2 was detected in large amounts in the placental tissues. Autopsy pathology showed positive viral staining in multiple fetal organs and the umbilical cord, but no histological abnormalities ascribable to viral infection. The authors stated that the placental abnormalities were consistent with intense vascular malperfusion which likely resulted in fetal death. A report from Russia described an immunocompromised mother with acute leukemia who developed COVID-19 and delivered a stillborn fetus at 20 weeks of gestation in which there was extensive placental damage causing placental insufficiency and fetal death [87].

In addition to individual case reports, there have been reports of cohorts of stillborn fetuses delivered to unvaccinated mothers with COVID-19 that have also demonstrated SARS-CoV-2 placental infection with microscopic findings of SARS-CoV-2 placentitis and fetal death that most probably resulted from placental insufficiency. Analysis of the placentas from the cluster of six perinatal deaths associated with the Alpha (B.1.1.7) variant of SARS-CoV-2 in County Cork (Ireland) [78] all demonstrated the same severe findings of increased or massive perivillous fibrin deposition, chronic histiocytic intervillositis and trophoblast necrosis—a triad of lesions termed SARS-CoV-2 placentitis by Watkins et al. [49]. Autopsy examination was performed in five cases that showed that all stillborns were anatomically normal with no microscopic evidence that SARS-CoV-2 had affected any fetal organ, and the authors ascribed the fetal deaths as due to placental insufficiency resulting from SARS-CoV-2 placentitis. In Sweden, Zaigham and colleagues reported five stillborn fetuses, including one set of twins, from mothers having COVID-19 infection [91]. The placentas of all stillborns were characterized by the findings of SARS-CoV-2 placentitis, and the authors concluded that the placental pathology-extensive fibrinoid deposits accompanied by villous necrosis characteristic of massive perivillous fibrin deposition accompanied by intervillositis accounted for the clinical outcomes of the stillborn fetuses. Furthermore, they believed that their findings indicated a rapid, progressive, and diffuse destruction of functional placental tissue leading to acute fetal distress and risk of intrauterine fetal demise within days from the onset of maternal COVID-19 infection. Debucs et al. in France [92] analyzed placentas from 50 unvaccinated pregnant mothers with COVID-19 which included five cases of intrauterine fetal demise occurring between 19 and 36 weeks 6 days of gestation. Maternal COVID-19 infection was diagnosed between 1 to 3 weeks prior to the diagnosis of intrauterine fetal demise, and all mothers had nonspecific mid symptoms and decreased fetal movements prior to delivery. All five stillborn placentas stained positively for SARS-CoV-2 using immunohistochemistry and had pathology findings of SARS-CoV-2 placentitis that occupied greater than 80 percent of the parenchyma including diffuse trophoblast necrosis, chronic histiocytic intervillositis and increased perivillous fibrin deposition. In addition, they all demonstrated intervillous hemorrhage and subchorionic hematomas. Three of the placentas were pathologically small, weighing in the 3rd percentile or below. Two of the fetuses underwent autopsy that revealed no significant pathology and negative staining for SASR-CoV-2 in visceral organs.

In the largest investigation of the cause of stillbirth and early neonatal death occurring with pregnant mothers having COVID-19, Schwartz and colleagues examined placentas infected with SARS-CoV-2 from 64 stillborn fetuses and four early neonatal deaths [93]. Because SARS-CoV-2 infection of the placenta is so uncommon, a multi-institutional study involving contributions from 12 countries was necessary to amass sufficient cases for analysis. In addition to the mothers having COVID-19, all 68 fetuses had intimate exposure to the virus because the placentas were confirmed to be positive for SARS-CoV-2 using immunohistochemistry, RNA in situ hybridization or PCR methods as previously recommended [42]. Stillbirth occurred at a mean gestational age of 30 weeks, with a modal value of 30 weeks 1 day. Delivery of the 64 stillbirths ranged from 15 weeks up to 39.2 weeks of gestation, and eight (13%) stillbirth cases were delivered at full term (>37 weeks gestation). The four cases of neonatal death were all delivered preterm, having a mean gestational age of 30.8 weeks and surviving an average of 3.5 days following delivery. All 68 placentas from this cohort demonstrated striking and severe pathology caused by elements of SARS-CoV-2 placentitis. The coexistence of chronic histiocytic intervillositis, increased fibrin deposition, and trophoblast necrosis was identified in 65/68 (97%) of placentas. Two of the three placentas that did not have all three of the constituents of SARS-CoV-2 placentitis, lacking chronic histiocytic intervillositis but had massive perivillous fibrin deposition and trophoblast necrosis. In one case, instead of massive perivillous fibrin deposition there were massive recent infarcts and decidual vessel thrombi present, together with trophoblast necrosis and chronic histiocytic intervillositis. Increased fibrin deposition was present in all 68 (100%) placentas from cases of stillbirth and neonatal death. Among the 68 placentas having increased fibrin there was massive perivillous fibrin deposition present in 63 (93%) cases, which was present together with trophoblast necrosis in all 63 cases (100%), and with chronic histiocytic intervillositis in 61 (98%). Chronic histiocytic intervillositis occurred in 66/68 (97%) of placentas. Among the 66 placentas having chronic histiocytic intervillositis, 62 of them (94%) had concurrent massive perivillous fibrin deposition. Villous trophoblast necrosis was present in all 68 (100%) placentas from stillbirths and neonatal deaths. In addition to SARS-CoV-2 placentitis, other abnormalities included intervillous thrombi/hemorrhages in 25 (37%) placentas, villitis in 22 (32%), maternal vascular malperfusion in 12 (18%), acute chorioamnionitis in 9 (13%), and fetal vascular malperfusion in 7 (10%) cases. There were 23 placentas that measured below the 10th percentile of weight stratified for gestational age. An important aspect of this study was the estimation of placental involvement with the destructive lesions of SARS-CoV-2 placentitis. This metric was calculated for each of 68 placentas to understand the impact of the pathology findings on placental function. The results were striking-the average placenta had 77.7% involvement from SARS-CoV-2 placentitis, a degree of placental damage and resulting malperfusion far exceeding the extent of placental involvement and destruction that is typically seen from infections with other TORCH agents. This level of placental damage severely impeded the delivery of sufficient oxygen and nutrients to the fetus to sustain life. In examining the results of this study, and in consideration of not only the destructive nature of the individual placental abnormalities of SARS-CoV-2 placentitis but also the occurrence of additional placental pathology findings including intervillous thrombi, villitis and maternal vascular malperfusion, it can be reasonably concluded that placental insufficiency was occurring together with fetal hypoxia which produced a hypoxic-ischemic fetal or neonatal demise. Among these 68 cases of stillbirth and neonatal death, there were no other significant potential etiologies besides placental insufficiency identified for perinatal demise from either a clinical or pathological perspective. The 30 autopsies described in this study demonstrated no evidence that the SARS-CoV-2 virus was causing stillbirth or neonatal death by inducing fetal somatic organ damage following placental infection and transplacental transmission. Instead, the tissue damage appeared to be confined to the placenta, where it was extensive and highly destructive, leading the authors to conclude that placental insufficiency was the probable cause of fetal and neonatal demise amongst these 68 cases. All of the pregnant women in this cohort were unvaccinated for COVID-19, raising the question of the potential significance of vaccination as a preventive strategy to avoid SARS-CoV-2 placentitis and stillbirth.

The findings from all of these placental pathology studies have clarified the probable mechanism of intrauterine demise for stillborn fetuses delivered to mothers with COVID-19. The syncytiotrophoblast is the most frequent placental cell type to become infected with SARS-CoV-2 [50], most likely as a result of virus reaching the placenta from the mother’s bloodstream during an episode of maternal viremia. SARS-CoV-2 placentitis and its 3 key components–increased fibrin deposition, trophoblast necrosis and chronic histiocytic intervillositis–appears to develop rapidly and worsen following initial placental infection. A combination of increasingly severe parenchymal ischemia caused by fibrin deposition and/or massive perivillous fibrin deposition and chronic histiocytic intervillositis obstructing maternal perfusion in the intervillous space, together with trophoblast necrosis, leads to progressive destruction of the placental parenchyma. The effects of SARS-CoV-2 placentitis, placental dysfunction and malperfusion produces fetal hypoxia, which is likely exacerbated by additional pathology findings such as placental hemorrhages, thrombohematomas, villitis, maternal vascular malperfusion, and in some cases, small placental size. The placental destruction from SARS-CoV-2 placentitis averages over three-quarters of the placental volume and effectively renders the placenta incapable of performing its function in oxygenating the fetus [93]. The ensuing placental insufficiency eventually results in hypoxic ischemic injury to the vital organs of the fetus, causing intrauterine demise. Autopsies performed on the fetuses in various studies have shown that fetal organ infection with SARS-CoV-2 is very uncommon, and even when present, is not associated with any significant tissue damage. However, in those cases where the placental damage is severe, it could be argued that that even when fetal organ infection from SARS-CoV-2 is present it would be almost irrelevant in causing fetal demise.

While these studies do not imply that vertical transmission of SARS-CoV-2 is never implicated in stillbirth, they do provide pathology-based data illustrating the mechanisms of death in selected groups of fetuses from mothers having COVID-19. Thus, unlike other viral, bacterial and parasitic TORCH agents that cause stillbirth following transplacental maternal-fetal transmission and direct infection and damage to fetal organs, SARS-CoV-2 can cause stillbirth even in the absence of fetal organ infection by producing placental insufficiency and hypoxic ischemic fetal injury.

## 5. Potential Importance of Vaccination of Pregnant Women to Reduce Risk of Viremia and Stillbirth

The finding that stillbirth in pregnant women with COVID-19 can result from placental infection and insufficiency has several clinical consequences. Similar to other viral infections that can undergo maternal-fetal transmission such as Zika virus, cytomegalovirus, rubella, Ebola virus and others [94], SARS-CoV-2 likely reaches the placenta through the maternal bloodstream as a result of viremia. In non-pregnant adults with COVID-19, viremia is highly unusual, transient and with low levels, occurring in approximately 1% of symptomatic individuals [95]. Although data on viremia in pregnant women with COVID-19 is scant, SARS-CoV-2 infection is uncommon and not associated with high levels of viremia [22,96]. Maternal viremia with SARS-CoV-2 has been described to occur in cases of SARS-CoV-2 placentitis and intrauterine fetal demise [97], and it is highly probable that reported cases of SARS-CoV-2 placentitis and stillbirth were the result of an episode of the virus circulating in the mothers’ bloodstream at some time during the pregnancy. In the 68 cases of stillbirth and early neonatal death from placental insufficiency arising from SARS-CoV-2 placentitis described by Schwartz et al. [93], as well as the six perinatal deaths from Ireland described by Fitzgerald et al. [78], the five stillborns from France reported by Debucs et al. [92], five stillborns from Sweden reported by Zaigham et al. [91], and others [98], the mothers were all unvaccinated for coronavirus.

Vaccines can be highly effective in reducing the pathogenic effects of viral infection that result from viremia [99,100]. The current vaccines to SARS-CoV-2 are designed to elicit a humoral and cell mediated immune response, preventing viremia and the COVID-19 syndrome [99,100]. Although it is not yet known to what degree the highly protective effects of COVID-19 vaccination result from cellular versus humoral immunity, it is clear that the highly efficacious mRNA vaccines produced by BioNTech and Moderna mRNA elicit a robust antibody response [101]. Thus, if viremia was reduced in concentration or duration, or even eliminated, through maternal vaccination then placental exposure to SARS-CoV-2 might potentially be reduced or avoided, decreasing the risk of stillbirth resulting from COVID-19 during pregnancy. The recent knowledge that stillbirth occurring in mothers with COVID-19 results from placental infection and insufficiency from SARS-CoV-2 placentitis emphasizes the importance of vaccination in pregnant women as a preventive strategy to avoid perinatal deaths.

## Data Availability

The data is available from publicly accessible repositories.
